# Effectiveness of Raw, Natural Medical *Cannabis* Flower for Treating Insomnia under Naturalistic Conditions

**DOI:** 10.3390/medicines5030075

**Published:** 2018-07-11

**Authors:** Jacob M. Vigil, Sarah S. Stith, Jegason P. Diviant, Franco Brockelman, Keenan Keeling, Branden Hall

**Affiliations:** 1 Department of Psychology, University of New Mexico, Albuquerque, NM 87131, USA; jdiviant@live.com; 2 Department of Economics, University of New Mexico, Albuquerque, NM 87131, USA; ssstith@unm.edu; 3 Morebetter Ltd., Washington, DC 20012, USA; Franco@releafapp.com (F.B.); Keenan@releafapp.com (K.K.); Branden@releafapp.com (B.H.)

**Keywords:** insomnia, *Cannabis*, marijuana, sleep, sleep disturbance, flower, cannabidiol, tetrahydrocannabinol, *C. indica*, *C. sativa*

## Abstract

**Background**: We use a mobile software application (app) to measure for the first time, which fundamental characteristics of raw, natural medical *Cannabis* flower are associated with changes in perceived insomnia under naturalistic conditions. **Methods**: Four hundred and nine people with a specified condition of insomnia completed 1056 medical cannabis administration sessions using the Releaf App^TM^ educational software during which they recorded real-time ratings of self-perceived insomnia severity levels prior to and following consumption, experienced side effects, and product characteristics, including combustion method, cannabis subtypes, and/or major cannabinoid contents of cannabis consumed. Within-user effects of different flower characteristics were modeled using a fixed effects panel regression approach with standard errors clustered at the user level. **Results**: Releaf App^TM^ users showed an average symptom severity reduction of −4.5 points on a 0–10 point visual analogue scale (SD = 2.7, d = 2.10, *p* < 0.001). Use of pipes and vaporizers was associated with greater symptom relief and more positive and context-specific side effects as compared to the use of joints, while vaporization was also associated with lower negative effects. Cannabidiol (CBD) was associated with greater statistically significant symptom relief than tetrahydrocannabinol (THC), but the cannabinoid levels generally were not associated with differential side effects. Flower from *C. sativa* plants was associated with more negative side effects than flower from *C. indica* or hybrid plant subtypes. **Conclusions**: Consumption of medical *Cannabis* flower is associated with significant improvements in perceived insomnia with differential effectiveness and side effect profiles, depending on the product characteristics.

## 1. Introduction

Nearly 50% of the adult population in the United States (US) experiences sleeping problems [1,2,3,4], with high rates of dissatisfaction over the effectiveness and potential side effects of conventional pharmaceutical sleep aid medications [5]. Prescription sleep aids, including antidepressants, benzodiazepines, gamma-aminobutyric acid (GABA) medications, and anti-psychotics, are associated with significant negative side effects and risks of dangerous drug interactions [6]. Over-the-counter (OTC) medications such as antihistamines, melatonin, and valerian, are generally less dangerous than prescription pharmaceuticals, but also less effective, and can also carry negative consequences (e.g., headaches, confusion, agitation), including residual effects (e.g., drowsiness, difficulty concentrating, and memory impairments) that can lead to secondary problem behaviors (e.g., lethargy, work absenteeism) [2,7,8,9,10,11]. First generation antihistamines (e.g., diphenhydramine, hydroxyzine, doxylamine succinate, and clemastine) act as anticholinergics. They generally score high on the anticholinergic cognitive burden scale. Alone or coupled with other anticholinergics, these medications, in addition to other classes of muscarinic antagonists, may contribute to an increased risk of developing dementia because the effects are cumulative and the body’s production of acetylcholine diminishes with age [12]. These circumstances may make people with insomnia willing to experiment with alternative sleep aid therapies, including medical cannabis, which is commonly used for treating insomnia [13,14,15] and becoming increasingly accessible due to expanding medical and now recreational cannabis reformation laws.

Clinical randomized controlled trials (RCTs) have historically used cannabis extracts and synthetic phytocannabinoid analogues for estimating the pharmacodynamic properties of cannabis used in vivo [16]. They generally show that while cannabis consumption can improve some sleep outcomes (e.g., latency to sleep, reduced REM sleep problem behaviors), it is often associated with negative (albeit, relatively minor) side effects, and the major phytocannabinoids, delta-9 tetrahydrocannabinol (THC) and cannabidiol (CBD), may have different effects at different stages of sleep [17,18,19,20,21,22]. The improved sleep outcomes associated with THC may be influenced by the putative interaction of the CB1 cannabinoid receptor with orexin receptors that are, in turn, targeted by sleep aid medications such as suvorexant [23]. Unfortunately, RCTs are poorly suited for assessing real-life patient decisions and capturing the phasic and inconsistent nature of the most common type of cannabis products used by millions of people daily, raw whole natural dried *Cannabis* flowers [16,24]. No study to date has measured the relative associations between THC and CBD contents and other basic characteristics of natural *Cannabis* flower (e.g., route of administration, cannabis subtypes) consumption and self-perceived feelings of insomnia in real-time under naturalistic conditions.

This is the first study to measure which fundamental attributes of commonly consumed dried *Cannabis* flower affect perceived insomnia levels and experienced side effects. We operationalize our research question using a mobile educational software application (app) [25] for recording how combustion method, cannabis subtypes, and major cannabinoid contents are associated with real-time measurements of subjective insomnia levels, prior to and following administration of cannabis, and the manifestation of myriad possible side effects from normative use in users’ natural environments.

## 2. Materials and Methods

### 2.1. Study Design

Institutional Review Board exemption was obtained from the University of New Mexico for this study, and the data were obtained from MoreBetter Ltd. (Washington, DC, USA), subject to a confidentiality agreement. This study uses self-collected data and user experiences recorded with the Releaf App^TM^ (version 1.4.1, Morebetter LTD, Washington, DC, USA) between June 2016 and May 2018. This mobile device educational software application was designed to track the effects of different types of medical cannabis products used under normal circumstances in natural environments, so that users can more optimally treat their underlying medical condition, with insomnia as one possibly treatable condition. Users of this version of the app consented through a privacy agreement for their anonymous data to be statistically analyzed and published in aggregate form. Prior to beginning each session, the user specifies the condition to be treated and the starting symptom level as well as a broad range of product characteristics. (The user interface for the Releaf App^TM^ is shown in Supplemental Figure S1). For the purpose of uniformity, we include only users of *Cannabis* flower. For analyses including THC and CBD, we omitted observations with labels indicated THC potencies greater than 35% or CBD potencies greater than 30%, because higher percentages do not occur naturally in flower.

### 2.2. Study Outcomes

Our study outcomes focus on symptom relief (reductions in insomnia symptom severity) and side effects. Product characteristic entry is voluntary, so the number of observations varies depending on the product characteristics included. Once a session has begun, the user can update their symptom level at any time. Symptom levels range from 0 (no detectable level) to 10 (most severe intensity level). In our analysis, we include only users reporting an initial symptom level of 1 or higher. Symptom Relief is measured as the starting symptom level minus the ending symptom level and ranges between −10 (maximum symptom relief) and 9 (minimum possible symptom relief). Our other outcome variables are any side effect reported by category (negative, positive, and context-specific) and percent of total available side effects in that category with 13 negative side effects, 19 positive side effects, and 10 context-specific side effects available for selection at any time during the session. (The side effects with categories are listed in Supplemental Table S1).

### 2.3. Statistical Analysis

We use ordinary least squares panel regressions to analyze the effects of product characteristics on symptom relief and side effect profiles. We regress symptom relief on the product characteristics separately. The sample size changes due to non-reporting of product characteristics. For our six separate side effect outcomes, we include all the product characteristics together for the sake of brevity. Because starting symptom levels are a strong predictor of symptom relief, partly mechanistically (e.g., higher starting symptoms enable greater possible symptom relief), we include the starting symptom level in all our regressions. Although the outcomes are [0, 1] in the “any” side effect regressions and [0, 1] in the “percent” side effect regressions, we use ordinary least squares for the sake of consistency across regression models with different outcomes. All regressions include time-invariant user fixed effects and standard errors are clustered at the user level to account for heteroskedasticity and arbitrary correlation at the user level. Analyses were conducted using Stata 13.1.

## 3. Results

Four hundred and nine users reported using flower to treat insomnia during 1056 sessions. In addition to the type of product, the user is prompted to report the combustion method (joint [13%], pipe [38%], and vape [49%]), plant subtypes (*C. Indica* [60%], *C. Sativa* [6%], and hybrid [33%]), and THC and CBD content (percentage of total weight). The mean THC level was 20% (SD = 5.39 percentage points) and the mean CBD level was 5.7% (SD = 5.44 percentage points). Starting symptom levels average 6.6 (SD = 2.1), while ending symptom levels average 2.2 (SD = 2.1). Mean symptom relief was −4.5 (standard deviation = 2.7, d = 2.10, *p* < 0.001). During the average session, users in our sample report 10% of negative side effects, 21% of positive side effects, and 24% of context-specific side effects. In addition, in 57% of sessions, users report at least one negative side effect, in 95% at least one positive side effect, and in 86% at least one context specific side effect. Table 1 presents complete descriptive statistics. Additionally, insomnia patients reported using 461 different strains. Among the most frequently used strains, there was wide variability in cannabinoid contents, with two *C. indica* strains with fairly high THC (around 20%) and high (20%) to moderate (7%) CBD potencies (“Granddaddy Purple” and “Northern Lights”), followed by two hybrid strains with similar amounts of THC but CBD potencies of less than 4% (“OG Kush” and “Blue Dream”).

Table 2 shows the results from regressing Symptom Relief on flower characteristics with each column representing a separate regression. When entered separately, the analyses show that only the product characteristic CBD percentage has a statistically significant effect on symptom relief; each additional percentage point of CBD contents is associated with a decrease of −0.04 (*p* < 0.01) in symptom severity levels. Starting symptom levels are important determinants of patient symptom relief with coefficients implying that once we restrict the sample to only those patients who reported CBD and THC, patients with low starting symptom levels experience worsening insomnia symptoms with consumption of *Cannabis* unless they are consuming sufficiently high percentages of CBD. When all product characteristics are included jointly, we find that smoking from a pipe or using a vaporizer is associated with greater symptom relief than smoking joints, and suggestive evidence that higher CBD levels are associated with greater symptom relief even after controlling for other characteristics of the flower consumed. The coefficient on *C. sativa* versus hybrid strains is large in magnitude at 2.481, but is statistically significant at only the 0.1 level.

**Figure 1:**
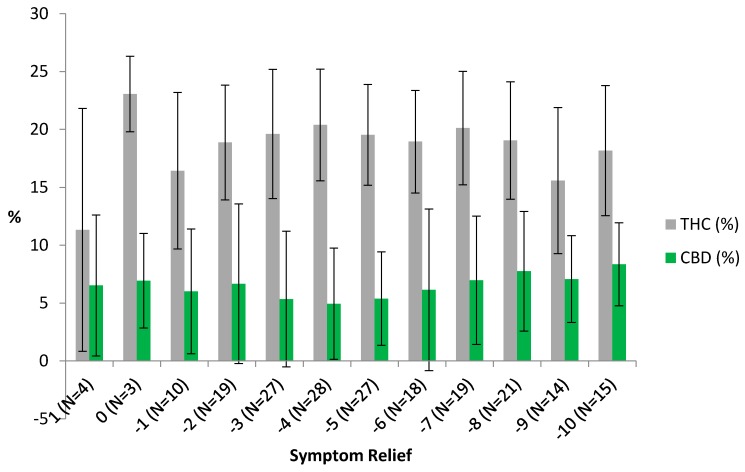
THC and CBD Levels by Symptom Relief Experienced. Symptom relief is measured as ending symptom level minus starting symptom level with −10 the maximum level of symptom relief. Error bars show the range of values within one standard deviation of the mean. No users who reported THC and CBD percentages reported symptom levels worsening by more than one unit. The number of sessions for each level of symptom relief is reported in parentheses with the sample restricted to those users reporting both CBD and THC potency levels, which range between 0% and 30% and 2% and 35%, respectively.

Figure 1 explores these relationships in greater detail in the raw data, depicting the mean THC and CBD percentages by level of symptom relief. The left hand (low symptom relief) side of the figure is likely fairly noisy due to small session counts, but the figure does suggest that non-linearities may exist in the general improvement in symptom relief with higher CBD levels and lower THC levels. Figure 1 also shows that THC potencies tend to be much higher than CBD potencies across all levels of symptom relief. This may indicate an interaction effect or that the optimal ranges may differ for the two cannabinoids.

In our regressions of side effects on product characteristics (Table 3), we find that product characteristics matter. In particular, *C. sativa* strains appear to be associated with more reporting of negative side effects, while vaping is associated with reduced reporting of negative side effects relative to consumption using joints and pipes. Individuals smoking joints appear to be the least likely to report positive or context-specific side effects. THC and CBD, however, do not appear to be associated with much variation in side effect reporting, except for a potential reduction in the extensive margin for context-specific side effects of 1.1 percentage points (*p* < 0.05). Higher starting symptoms are associated with increased negative side effects on both the extensive (any reported) and intensive (percent reported) margins as well as the reporting of a higher percent of positive side effects. Based on the R-squared, the product characteristics, individual user fixed effects and starting symptoms do a better job of explaining variation in positive and context-specific side effects than negative side effects.

## 4. Discussion

Given how important quality sleep is for optimizing mental and physical wellbeing, it is alarming how pervasive sleep disturbances are throughout society [2,3,4]. The limited effectiveness and risk of undesirable and potentially dangerous side effects of conventional pharmaceutical sleep aids [6,7] result in nearly 50% dissatisfaction rates [5]. Hence, it is not surprising why people with sleep disturbances commonly report regular experimentation with multiple types of sleep aids [26], including alcohol and *Cannabis*. Our results showed that on average, Releaf App^TM^ users experienced a statistically and clinically significant improvement (−4.5 points on a 0–10 point scale) in perceived insomnia levels. However, products made with *C. sativa* were associated with less symptom relief and more negative side effects than products made from *C. indica* or hybrid plant subtypes. Use of pipes and vaporizers was associated with greater symptom relief and more positive and context-specific side effects as compared to the use of joints, while vaporization was also associated with lower negative effects. CBD potency levels were associated with greater symptom relief than were THC levels, but the cannabinoid contents were generally not associated with differential reported side effects. 

The current results are consistent with survey-based studies showing increasing reported usage of cannabis for treating insomnia in healthy people and patients with other primary health conditions [13,14,15], and a patient preference for high CBD products [13,18,27]. In comparison to conventional prescription pharmaceutical sleep aids, CBD is generally believed to be much safer and often is described as non-psychoactive [16]. Prescription sleep aids in contrast, namely antidepressants (e.g., trazodone, amitriptyline, and doxepin), benzodiazepines (e.g., diazepam and lorazepam), gamma-aminobutyric acid (GABA) medications (zolpidem and eszopiclone), and anti-psychotics (aripiprazole, olanzapine, quetiapine and risperidone) are associated with significant clinical drawbacks [6] and heightened risk of morbidity [28,29,30,31]. The phytocannabinoid family of CBDs are known to differ from other cannabinoids such as THC in several ways, including having little affinity to CB1 receptors, serving as an antagonist to the effects of THC, and functioning as anti-inflammatory and immuno-suppressant agents [32,33]. Orexin antagonists, such as suvorexant [34], as well as nemorexant and lemborexant, which are currently in phase 3 clinical trials, are all dual antagonists of the orexin OX1 and OX2 receptors. They are being investigated for their potential use in treating sleep disorders. OX1 and OX2 receptors regulate several functions that overlap with cannabinoids, such as pain, wakefulness, and sleep. Both receptor types can form homo- and heterodimers with one-another and with CB1 receptors; however, orexin potentiation of CB1 signaling may result from orexin-promoted 2-AG (2-arachidonoyl glycerol, a native ligand of CB1 cannabinoid receptors) production and not necessarily from orexin-CB1 heterodimerization [23,35]. The activation of OX1 and OX2 receptors each modulate the effects induced by cannabinoids in different ways [36]. Whereas THC is a partial agonist of the CB1 receptor, CBD has a very low affinity for the CB1 receptor and instead acts as an indirect antagonist [37].

However, the fact that our results did not seem to show a clear relationship between THC or CBD and symptom relief suggests that other cannabinoid chemical(s) (e.g., cannabinols) and terpenes could contribute to changes in sleep experiences. Cannabinoid and terpene profiles vary across strains and we did find that the most frequently used cannabis strains for insomnia treatment were quite distinct in their chemotypic characteristics, highlighting the range of products and associated interactions among sub-compounds across products used by patients even just within flower. Therapeutically, cannabis consumption may also alleviate primary symptoms such as pain and anxiety, which are associated with sleep disturbances [16]. Unfortunately, due to cannabis’ continued Schedule I status and associated barriers to conducting medical cannabis research [24], no practical, naturalistic investigations have been completed on how patient-managed phytocannabinoid consumption affects discrete mechanisms (e.g., ventrolateral preoptic nucleus activation, memory consolidation) and other basic characteristics (e.g., sleep stages, circadian rhythm) involved in normal and aberrant sleep patterns.

Despite the novelty and practical implications of our findings, the observational nature of the research design had unavoidable drawbacks, most notably the absence of a comparison group, which could have resulted in overestimation of the effectiveness of cannabis if unsatisfied users chose not to use the Releaf App^TM^, or underestimation of cannabis’ effectiveness if users choose not to use the app as a result of accomplished satisfaction with product choices and their effects. It is also possible that the Releaf App^TM^ affected how users experience cannabis’ effects, and future research will benefit from examining both the effectiveness and influence of using electronic technology for patient medication management and monitoring. Small sample sizes could have also led to under-powered analyses, i.e., that other product characteristics matter but our sample is too small to pick them up at standard levels of statistical precision. Our study was also limited in the amount of information obtained by users and did not include detailed demographic characteristics, pre-app experience using cannabis, other types of sleep therapies, or type of sleep disorder. Finally, the study was limited to the accuracy of the product characteristics displayed on labels of the products consumed in the study, and there is a common problem of inaccurate (e.g., inflated) labeling practices in the medical cannabis industry in the U.S. [38].

Notwithstanding these limitations, this is the first study to measure how fundamental properties of self-directed *Cannabis* flower consumption affect immediate symptom relief from insomnia within users’ natural environments. Although no U.S. state has legalized medical cannabis for the treatment of sleep disorders, our results show that consumption of *Cannabis* flower is associated with significant improvements in perceived insomnia with differential effectiveness and side effect profiles. The widespread apparent use of cannabis as a sleep aid underscores the importance of further medical research regarding its risk-benefit profile and the effectiveness of cannabis as a substitute for other substances, including alcohol, over-the-counter and prescription sleep aids, and scheduled medications (e.g., opioids and sedatives) [14,39,40,41], many of which are used in part as sleep aids.

## Figures and Tables

**Table 1:** Descriptive Statistics.

Variable	Mean	Std. Dev	Minimum	Maximum
Panel A: Subtypes (983 sessions, 378 users)				
Hybrid	0.33	0.47	0	1
*C. indica*	0.60	0.49	0	1
*C. sativa*	0.06	0.24	0	1
Panel B: Combustion Method (996 sessions, 385 users)				
Joint	0.13	0.34	0	1
Pipe	0.38	0.48	0	1
Vape	0.49	0.50	0	1
Panel C: THC (353 sessions, 143 users)				
% THC	0.19	0.54	0.02	0.35
THC < 10%	0.05	0.22	0	1
THC 10–19%	0.50	0.50	0	1
THC 20–34%	0.45	0.50	0	1
Panel D: CBD (119 sessions, 281 users)				
% CBD	0.60	0.54	0	0.30
CBD 0%	0.19	0.39	0	1
CBD 1–9%	0.51	0.50	0	1
CBD 10–34%	0.30	0.46	0	1
Panel E: Outcome and Control Variables (1056 sessions, 409 users)				
Symptom Change	−4.5	2.7	−10	9
Starting Symptom Level	6.6	2.1	1	10
Ending Symptom Level	2.2	2.1	0	10
Panel F: Side Effects (1215 sessions, 359 users)				
Any Negative Side Effect	0.57	0.50	0	1
% of Negative Side Effects	0.10	0.13	0	1
Any Positive Side Effect	0.95	0.23	0	1
% of Positive Side Effects	0.21	0.15	0	1
Any Context-Specific Side Effect	0.86	0.35	0	1
% of Context-Specific Side Effects	0.24	0.19	0	1

Note: The different types of variables are grouped in Panels A through F, with the number of sessions and users for whom that information is available listed in parentheses. For each variable in each panel, we report the mean, standard deviation, minimum, and maximum values for that variable. Our variables are all dichotomous {0, 1} with the exception of THC, CBD, and the % of side effects variables, which are percentages range from 0 to 1, and the symptom relief measures, which are measured on a 0 to 10 scale with 0 being no discernable symptom level and 10 extreme symptom severity. Symptom change is measured as the ending symptom minus the starting symptom level for a range between −10 and 9. Starting symptom levels are restricted to range from 1 to 10, while ending symptom levels range from 0 to 10. Nineteen positive, thirteen negative, and ten context-specific side effects were available for selection in Panel F.

**Table 2:** Effects of Product Characteristics on Symptom Relief—Regression Results.

Variable	(1)	(2)	(3)	(4)
Panel A: Subtypes, omitted category = hybrid				
*C. indica*	−0.227			0.176
	(0.214)			(0.220)
*C. sativa*	−0.214			2.481 *
	(0.492)			(1.445)
Panel B: Combustion Method, omitted category = joint				
Pipe		−0.715		−1.686 **
		(0.563)		(0.758)
Vape		−0.823		−1.560 **
		(0.583)		(0.782)
Panel C: THC and CBD				
THC (%)			−4.759	−4.280
			(2.978)	(3.761)
CBD (%)			−3.828 ***	−5.232 *
			(1.121)	(2.841)
Starting Symptom Level	−0.763 ***	−0.781 ***	−0.951 ***	−0.873 ***
	(0.058)	(0.056)	(0.095)	(0.092)
Constant	0.603 *	1.318 **	2.599 ***	3.100 ***
	(0.362)	(0.617)	(0.920)	(1.115)
Observations	983	996	205	195
R-squared	0.341	0.337	0.562	0.613
Number of users	378	385	90	83

Notes: Each column represents a separate regression. Regressions control for individual user fixed effects. *C. indica* and *C. sativa* are relative to Hybrid, and Pipe and Vape are relative to Joint. Standard errors are clustered at the user level. *** *p* < 0.01, ** *p* < 0.05, * *p* < 0.1.

**Table 3:** Effects of Product Characteristics on Side Effects—Regression Results.

Variable	(1)	(2)	(3)	(4)	(5)	(6)
	Negative	% of Negative	Positive	% of Positive	Context-Specific	% of Context-Specific
*C. indica*	−0.034	0.001	0.037	−0.035 **	−0.004	−0.013
	(0.026)	(0.011)	(0.036)	(0.015)	(0.039)	(0.022)
*C. sativa*	0.478 ***	0.105 ***	−0.349	−0.002	−0.236	−0.018
	(0.152)	(0.028)	(0.277)	(0.088)	(0.309)	(0.131)
Pipe	−0.042	−0.044	0.025	0.116 **	0.917 ***	0.252 ***
	(0.122)	(0.038)	(0.022)	(0.050)	(0.075)	(0.050)
Vape	−0.484 ***	−0.067 *	0.034	0.135 ***	0.993 ***	0.248 ***
	(0.144)	(0.037)	(0.026)	(0.049)	(0.083)	(0.063)
THC (%)	−0.136	−0.002	0.031	−0.200	0.808	0.506
	(0.810)	(0.093)	(0.077)	(0.304)	(0.586)	(0.455)
CBD (%)	0.525	0.239	0.163	−0.008	−1.101 **	−0.006
	(1.263)	(0.159)	(0.142)	(0.154)	(0.496)	(0.241)
Starting Symptom Level	0.074 ***	0.012 ***	−0.002	0.012 ***	0.030	0.004
	(0.025)	(0.004)	(0.002)	(0.004)	(0.021)	(0.009)
Constant	0.388	0.035	0.955 ***	0.084	−0.297 *	−0.105
	(0.329)	(0.055)	(0.035)	(0.092)	(0.155)	(0.096)
Observations	170	170	170	170	170	170
R-squared	0.128	0.165	0.382	0.123	0.355	0.101
N Users	70	70	70	70	70	70

Notes: Each column represents a separate regression. Regressions control for individual user fixed effects. *C. indica* and *C. sativa* are relative to Hybrid and Pipe and Vape are relative to Joint. Standard errors are clustered at the user level. *** *p* < 0.01, ** *p* < 0.05, * *p* < 0.1.

## Supplementary Materials

The following are available online at http://www.mdpi.com/2305-6320/5/3/75/s1, Table S1: Categories and Frequency of Side Effect Reporting, Figure S1: Releaf App^TM^ User Interface.
