# Supplementary material for: Effectiveness of Raw, Natural Medical Cannabis Flower for Treating Insomnia under Naturalistic Conditions
Source: Medicines (Basel). 2018 Jul 11;5(3):75. doi: 10.3390/medicines5030075 (PMC6164964; doi:10.3390/medicines5030075)
Supplement: Supplementary file 1 [file medicines-05-00075-s001.pdf]

Supplementary Materials: Effectiveness of Raw, Natural Medical Cannabis Flower for Treating Insomnia under Naturalistic Conditions

Jacob M. Vigil, Sarah S. Stith, Jegason P. Diviant, Franco Brockelman, Keenan Keeling and Branden Hall

Table S1. Categories and Frequency of Side Effect Reporting.

Side Effect	% Sessions Reporting	Category
Foggy	25%	Negative
Dry Mouth	23%	Negative
Forgetful	12%	Negative
Unmotivated	12%	Negative
Dizzy	10%	Negative
Red Eyes	9%	Negative
Restless	9%	Negative
Scattered	9%	Negative
Anxious	7%	Negative
Confused	4%	Negative
Headache	4%	Negative
Paranoid	3%	Negative
Irritable	3%	Negative
Relaxed	72%	Positive
Peaceful	54%	Positive
Comfy	49%	Positive
Dreamy	47%	Positive
Chill	37%	Positive
Happy	19%	Positive
Reflective	18%	Positive
Great	17%	Positive
Light	16%	Positive
Tuned	13%	Positive
Focused	11%	Positive
Grateful	11%	Positive
Optimistic	11%	Positive
Clear	9%	Positive
Frisky	7%	Positive
Creative	6%	Positive
Productive	4%	Positive
Energetic	4%	Positive
Active	2%	Positive
Sleepy	56%	Context-Specific
High	45%	Context-Specific
Couchlocked	28%	Context-Specific
Thirsty	26%	Context-Specific
Tingly	20%	Context-Specific

Hungry	16%	Context-Specific
Distracted	12%	Context-Specific
Silly	7%	Context-Specific
Visuals	5%	Context-Specific
Talkative	4%	Context-Specific

Notes: Negative, Positive, and Context-Specific side effects are listed by category and ordered by the percent of sessions reporting that side effect in the data.

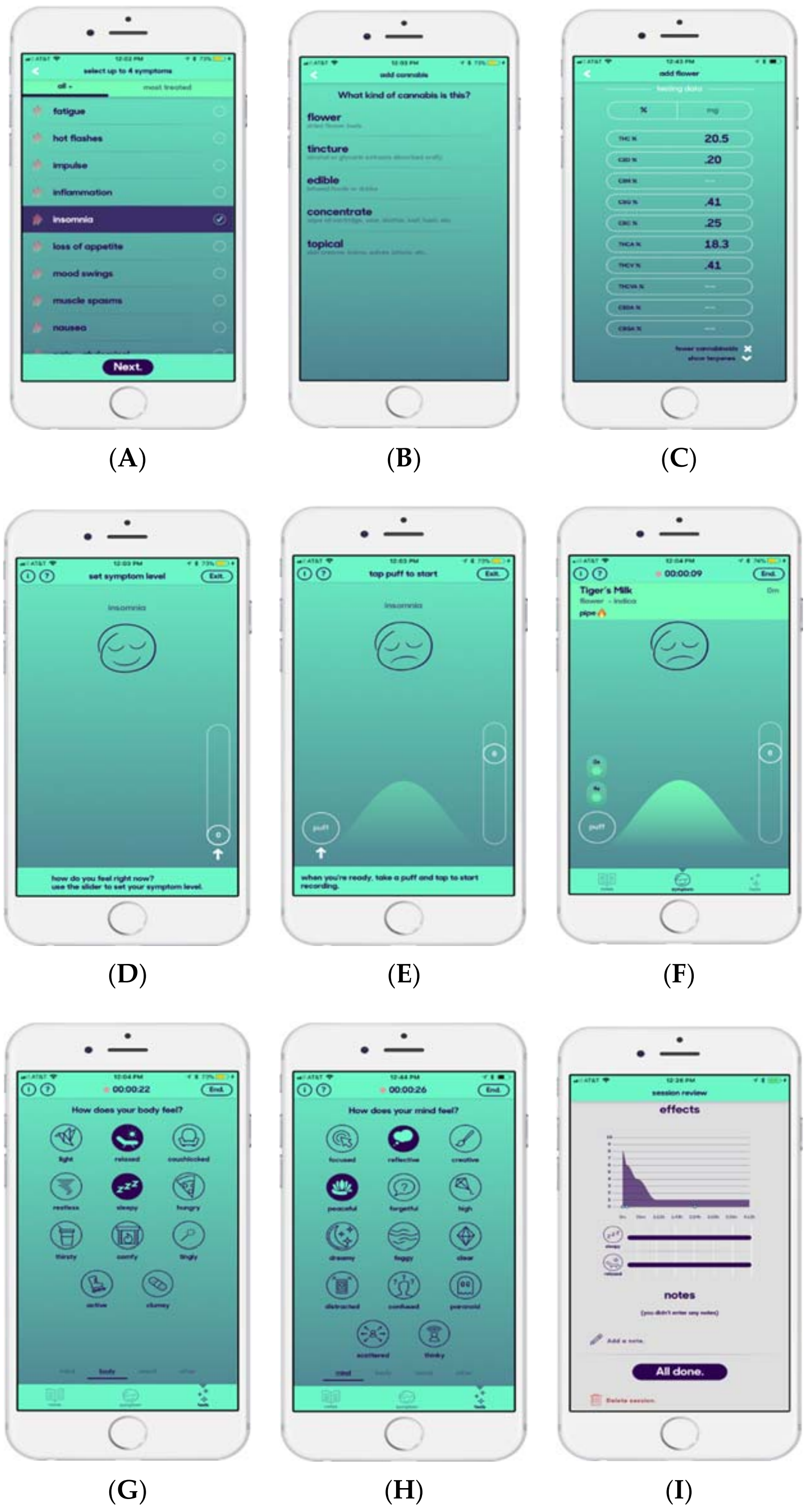

Figure 1. Releaf App™ User Interface. Notes: The panels show the user interface for: Indicating primary health condition (**A**); Selecting cannabis product type (**B**); Indicating *Cannabis* flower characteristics (**C**); Indicating baseline insomnia severity level (**D**); Directing the user to administer their medicine (**E**); Indicating post-consumption insomnia severity levels (**F**); Indicating body-associated side effects (**G**); Indicating mind-related side effects (**H**); Describing the user with feedback on their consumption session (**I**).
